# The Risk of Fractures in Primary Hyperparathyroidism: A Meta‐Analysis

**DOI:** 10.1002/jbm4.10482

**Published:** 2021-03-16

**Authors:** Niya Narayanan, Rajan Palui, Chandhana Merugu, Sitanshu Sekhar Kar, Sadishkumar Kamalanathan, Jayaprakash Sahoo, Sandhiya Selvarajan, Dukhabandhu Naik

**Affiliations:** ^1^ Department of Endocrinology Jawaharlal Institute of Postgraduate Medical Education and Research (JIPMER) Puducherry India; ^2^ Department of Preventive and Social Medicine Jawaharlal Institute of Postgraduate Medical Education and Research (JIPMER) Puducherry India; ^3^ Department of Clinical Pharmacology Jawaharlal Institute of Postgraduate Medical Education and Research (JIPMER) Puducherry India

**Keywords:** BONE MINERAL DENSITY, NONVERTEBRAL FRACTURE, RADIUS FRACTURE, TOTAL FRACTURE, VERTEBRAL FRACTURE

## Abstract

Primary hyperparathyroidism (PHPT) is a common metabolic bone disease affecting 1% of the adult population. Patients with PHPT have reduced BMD, especially at the cortical bone. However, studies evaluating its impact on fracture risk have shown contradictory results. In an effort to further inform fracture risk for this patient population, a meta‐analysis of studies of fracture in patients with PHPT compared with a control population was undertaken. Articles were searched in PubMed/MEDLINE, Excerpta Medica, Cochrane Central Register of Controlled Trials, Latin American and Caribbean Health Sciences Literature, and Web of Science bibliographic databases. The meta‐analysis included 17 studies involving 3807 PHPT cases and 11,908 controls. The primary outcome was to determine the risk of vertebral fracture (VF), nonvertebral fracture, hip fracture, distal radius fracture, and total fracture (TF) among patients with PHPT in comparison with a control population. BMD (lumbar spine, femoral neck, total hip, and distal radius) and serum 25‐hydroxy vitamin D level, as well as possible predictors of VF as secondary outcomes were assessed. From this meta‐analysis, it was found that there was a significantly increased risk of VF (risk ratio [RR], 2.57; 95% CI, 1.3–5.09; *p* = 0.007) and TF (RR, 1.71; 95% CI, 1.48–1.97; *p* < 0.00001) in patients with PHPT. There was a significant decrease in BMD in patients with PHPT versus controls at all four sites. Older age, longer duration since menopause, and lower BMD at lumbar spine and distal radius were predictors for VF. To conclude, patients with PHPT had a significantly higher risk for VF and TF in comparison with controls. © 2021 The Authors. *JBMR Plus* published by Wiley Periodicals LLC on behalf of American Society for Bone and Mineral Research.

## Introduction

Primary hyperparathyroidism (PHPT) is a common disorder of bone and mineral metabolism caused by excessive secretion of PTH from the parathyroid glands. Classically, PHPT is characterized by hypercalcemia and concentrations of PTH, which is either elevated above the normal range or inappropriately normal in the context of hypercalcemia.^(^ ^1^ ^)^ PHPT is caused by solitary parathyroid adenomas in 80% to 85% of cases.^(^ ^2^ ^)^ It is more common among women with a women‐to‐men ratio of 3:1 to 4:1.^(^ ^2^ ^)^ PHPT is seen in 1% of the adult population; its prevalence increases to 2% in adults above 55 years of age in Western populations.^(^ ^3^ ^)^ The incidence of PHPT varies from approximately 0.4 to 82 cases per 100,000.^(^ ^4^ ^)^ Its incidence is highest among Blacks, followed by Whites, whereas the rates for Asians, Hispanics, and other races are lower than that of a White populace.^(^ ^5^ ^)^

In the 1930s, Albright first described the hypercalcemic state caused by PHPT as a disease of “bones, stones, moans, and groans.”^(^ ^6^ ^)^ With the advent of automated biochemical screening, the clinical presentation of PHPT has changed. Mild hypercalcemia detected incidentally in asymptomatic older subjects is presently the most common presentation worldwide. On the contrary, PHPT, presenting in later stages with classical involvement such as the skeleton and renal system, is limited primarily to developing countries like India, where routine biochemical screening is not practiced, and vitamin D deficiency is endemic. The majority (95%) of the histopathologically proven patients with PHPT of a nationwide registry from India were symptomatic as mentioned by Bhadada et al.^(^ ^7^ ^)^ The mean age of presentation of PHPT in India is also a decade earlier compared with patients from Western countries.^(^ ^7^ ^)^

Patients with PHPT typically have high bone remodeling as assessed by bone turnover markers caused by continuous exposure to high PTH levels.^(^ ^8^ ^)^ The pattern of bone loss in PHPT leads to certain expectations of fracture risk. With a reduction in cortical BMD and relative preservation of trabecular BMD, patients with PHPT would be expected to be at increased risk for distal radial fracture (RF) and reduced risk for vertebral fracture (VF).^(^ ^2^ ^)^ Overall, PHPT accounts for approximately 3% of newly diagnosed pathological fractures.^(^ ^9^ ^)^ Fracture is one of the common comorbidities associated with symptomatic PHPT. Risk of fracture is a major determinant in deciding the need for surgery among patients with PHPT; thus, it is important to evaluate the accurate risk of fractures in them. However, findings from various studies reporting the prevalence of fracture in PHPT are heterogeneous and inconsistent.^(^ ^8^ ^)^ Meta‐analyses done on the effect of parathyroidectomy on the fracture incidence in patients with PHPT are there in the literature.^(^ ^10^ ^)^ However, there has been no meta‐analysis comparing fracture incidence in patients with PHPT with apparently healthy controls. Thus, the primary aim of our study was to measure the risk of fracture in patients with PHPT in comparison with a control population. The secondary outcomes are to compare BMD between PHPT and a control population and to assess the possible risk factors for VF in PHPT.

## Materials and Methods

This meta‐analysis was conducted in accordance with the predefined protocol registered in International Prospective Register of Systematic Reviews (PROSPERO; registration no. CRD42020156236).^(^ ^11^ ^)^ Reporting of the meta‐analysis was done as per the Preferred Reporting Items for Systematic Reviews and Meta‐Analyses (PRISMA) guidelines.^(^ ^12^ ^)^ As this study was done with the data already available in the published literature, no separate ethics approval was required.

### Search strategy

We searched the following electronic bibliographic databases from their dates of inception through March 31, 2019 to find relevant articles: PubMed/MEDLINE, Excerpta Medica (EMBASE), Cochrane Central Register of Controlled Trials, Latin American and Caribbean Health Sciences Literature (LILACS), and Web of Science (WOS). We also searched for unpublished studies in the US National Institutes of Health, Department of Health and Human Services Trials Registry^(^ ^13^ ^)^ and the World Health Organization (WHO) International Clinical Trials Registry Platform (ICTRP).^(^ ^14^ ^)^ The search terms used were “primary hyperparathyroidism,” “PHPT,” and “fracture.” The details of the search strategy are given in Supplementary Information Table S1. Only studies published in English were included in this review. References of the included studies were also manually searched for relevant articles. Two authors (NN and CM) independently performed a literature search; any disagreement was solved after discussion with a senior author (RP). We contacted the corresponding authors of the selected articles to clarify the published data whenever it was necessary.

### Inclusion and exclusion criteria

All clinical studies (randomized control trials [RCTs], cohort, case–control, or cross‐sectional design), analyzing fracture in patients with PHPT in comparison with a control population where the prevalence of fracture was available or had sufficient data to calculate it, were included in the analysis. If there was more than one publication from the same cohort of patients, the study with longest follow‐up or maximum number of participants was included in our meta‐analysis. Studies that included patients with secondary/tertiary hyperparathyroidism were excluded from analysis. Articles such as case reports, case series, conference presentations, commentaries, editorials, meta‐analyses, or letters were also excluded from our study. Two of the authors (NN and CM) independently identified potential eligible studies for selection after screening the abstracts. Subsequently, full texts of the selected studies were reviewed. In case of any discrepancy, consensus about eligibility of the studies was reached after discussion with a senior author (JPS).

### Data extraction

Data from the included studies were extracted in a standardized predesigned EXCEL format. The following study characteristics were extracted from the selected studies: the author, country, study design, number of patients or controls, and the characteristics of the control population. The following parameters were extracted from individual studies: age, gender, duration (year/s) since menopause, BMI, prevalence of fracture, PTH (pg/mL), calcium (mg/dL), phosphorus (mg/dL), bone‐specific alkaline phosphatase (μg/L), creatinine (mg/dL), 25‐hydroxy vitamin D (ng/mL), 1,25‐dihydroxyvitamin D (pmol/L), osteocalcin (ng/mL), BMD at the lumbar spine (gm/cm^2^), BMD at the femoral neck (gm/cm^2^), BMD at the total hip (gm/cm^2^), and BMD at the distal radius (gm/cm^2^). All the data were extracted from text, tables, or figures of the article as needed. For missing data, the corresponding authors of the included studies were contacted. NN and CM extracted the data, and any disagreements arising regarding the data extraction were resolved after discussion with a third author (SK).

### Risk of bias

The study quality of the included studies was independently assessed by DBN, SS, and SSK using the Methodological Index for Non‐Randomized Studies (MINORS) scale.^(^ ^15^ ^)^ The study was considered to be an ideal study if the global score was 24 or more.

### Specifications of outcomes

The primary outcome of our meta‐analysis was to determine the risk of VF, nonvertebral fracture (NVF), hip fracture (HF), RF, and total fracture (TF) among patients with PHPT in comparison with a control population. The number of fractures included all fractures including fragility fractures. The classification of fracture was defined as per the categorization done by the primary authors of the included articles. The TF is defined as per the discretion of the primary author; it includes both vertebral and all NVFs. The RF is defined as any fracture in the distal portion of the radius bone. The secondary outcome of our meta‐analysis was to compare BMD (lumbar spine, femoral neck, total hip, and distal radius) and the serum 25‐hydroxy vitamin D level between the patients with PHPT and the control population. We also did a subgroup analysis for VF, NVF, and TF according to common risk factors like gender and disease severity. The disease severity was considered as symptomatic and mild as defined by the authors. Subgroup analysis was not possible for HF and RF because of nonavailability of the data. In addition, we also analyzed possible risk factors of VF in the PHPT by comparing patients with and without VF.

### Statistical analysis

The effect size of the primary outcome of our study was analyzed by the difference between fracture rate among patients with PHPT and the control population in terms of risk ratio (RR) with 95% CI using the Mantel–Haenszel method. The number of persons with fracture from the included studies was used for the calculation of the effect size (RR). The weighted mean difference (MD) with 95% CI was used to compare BMD and serum 25‐OH vitamin D levels between the two groups. We also calculated the MD with 95% CI for various continuous variables between patients with PHPT with or without VF to look for possible risk factors. The random effect model was used for statistical analysis throughout the study because there was a possibility that the true effect size varied from study to study as the studies had different backgrounds. A sensitivity analysis was done wherever it was required. The *I* ^2^ statistic and Cochrane Q test were used for heterogeneity analysis. Heterogeneity was considered low, moderate, and high based on *I* ^2^ values of 25% to 50%, 50% to 75%, and >75%, respectively.^(^ ^16^ ^)^ A *p* value <0.05 was considered statistically significant throughout the study. Publication bias was assessed by funnel plot, Egger's regression test, and Begg's test. Statistical analysis was done using Review Manager (Revman) 5.3 software^(^ ^17^ ^)^ and comprehensive meta‐analysis software.

## Results

### Search and selection of studies

**Fig 1:**
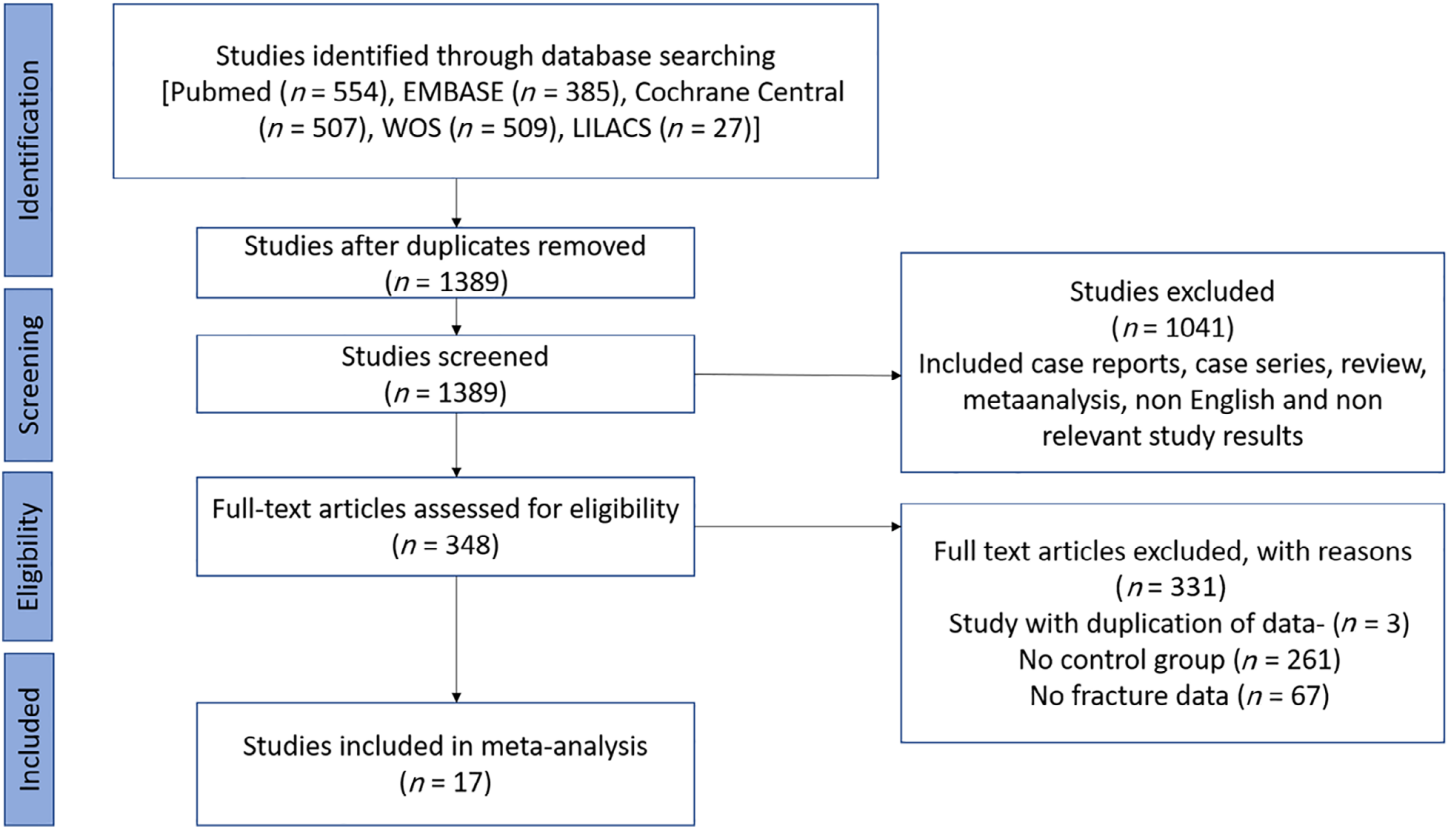
Preferred Reporting Items for Systematic Reviews and Meta‐Analyses (PRISMA) flow chart of the study selection process. EMBASE Excerpta Medica database; LILACS, Latin American and Caribbean Health Sciences Literature; WOS, Web of Science

A total of 1389 relevant articles were identified after a bibliographic search, of which 348 studies were assessed in detail for the fulfillment of eligibility criteria. Among them, 261 articles were excluded based on the unavailability of a control population. Similarly, 67 studies had no fracture data in either the controls or patients with PHPT. Three studies were excluded because of duplication of data.^(^ ^18^, ^19^, ^20^ ^)^ Finally, 17 studies were included for our meta‐analysis.^(^ ^21^, ^22^, ^23^, ^24^, ^25^, ^26^, ^27^, ^28^, ^29^, ^30^, ^31^, ^32^, ^33^, ^34^, ^35^, ^36^, ^37^ ^)^ A summary for the selection of eligible studies is depicted in the PRISMA‐flow chart (Fig. 1).

### Study characteristics and quality assessment

Our meta‐analysis included 17 studies involving 3807 PHPT cases and 11,908 controls. Two studies included only patients with mild PHPT.^(^ ^23^, ^33^ ^)^ The controls were either screened from the healthy population or patients attending the outpatient department of the hospital for unrelated conditions; they were age‐ and gender‐matched in most of the studies. A summary of the characteristics of the included studies is provided in Table 1. Ten of the studies were conducted in Europe, five in the United States, and two in Asia. The majority of the studies were cohort (seven retrospective and eight prospective cohort studies);^(^ ^21^, ^22^, ^23^, ^24^, ^25^, ^26^, ^27^, ^28^, ^30^, ^31^, ^32^, ^33^, ^34^, ^35^, ^36^ ^)^ the other two studies were cross‐sectional^(^ ^29^ ^)^ and case control.^(^ ^37^ ^)^ The studies were published between 1975 and 2019. The assessment of the study quality based on the risk of bias is summarized in Supplementary Information Table S2. The MINORS scale for the included studies are in the range of 7 through 20, suggestive of a less than ideal study.

**TABLE 1:** Summary of Study Characteristics

Sl No.	Study reference	Year	Type of study	Country	Control	Participants(N) (PHPT, control)	Female(%) (PHPT, control)	Age (mean, y)
1	Dauphine et al^(^ ^21^ ^)^	1975	Retrospective cohort	USA	Laminectomy subjects	224, 479	66.5, 35.3	61.6, 59
2	Kochersberger et al^(^ ^22^ ^)^	1987	Retrospective cohort	USA	Age matched (±5 y) Cholecystectomy subjects	191, 192	79, 74	61^a^,65^a^
3	Wilson et al^(^ ^23^ ^)^	1988	Retrospective cohort	USA	Historical cohort	174, 209	80.5, 100	62, NA
4	Larsson et al^(^ ^24^ ^)^	1989	Retrospective cohort	Sweden	Healthy postmenopausal subjects matched for age, YSM, & BMI	39, 34	100, 100	68, 68
5	Melton et al^(^ ^25^ ^)^	1992	Retrospective cohort	USA	Age (±2 y) & gender‐matched subjects	90, 90	73.3, 73.3	58.5, 58.7
6	Kenny et al^(^ ^26^ ^)^	1995	Retrospective cohort	USA	Healthy postmenopausal subjects matched for age, height, weight, & age at menopause	46, 44	100, 100	68.9, 67.4
7	Vestergaard et al^(^ ^27^ ^)^	2000	Retrospective cohort	Denmark	Age & gender matched	674, 2021	74.3, NA	58.2, 58.2
8	Minisola et al^(^ ^28^ ^)^	2002	Prospective cohort	Italy	Normal postmenopausal women	33, 27	100, 100	60.8,60.3
9	Kaji et al^(^ ^29^ ^)^	2005	Cross sectional	Japan	Women screened for osteoporosis	116, 716	100, 100	60, 61.2
10	De Geronimo et al^(^ ^30^ ^)^	2006	Prospective cohort	Italy	Postmenopausal healthy matched for age, YSM, & BMI	98, 89	100, 100	61.4, 60.6
11	Vignali et al^(^ ^31^ ^)^	2009	Prospective cohort	Italy	Postmenopausal healthy matched for age (±2 y) & YSM (±5 y)	150, 300	100, 100	61, 61
12	Hansen et al^(^ ^32^ ^)^	2010	Prospective cohort	Denmark	Age matched	27, 27	100, 100	60^a^, 60^a^
13	Yu et al^(^ ^33^ ^)^	2011	Prospective cohort	UK	Age & gender matched	1424, 7120	70.3, 70.3	68.3, 68.3
14	Eller‐Vainicher et al^(^ ^34^ ^)^	2013	Prospective cohort	Italy	Postmenopausal women and eugonadal men	92, 98	80.4, 82.7	62.7, 62.1
15	Romagnoli et al^(^ ^35^ ^)^	2013	Prospective cohort	Italy	Postmenopausal women matched for age, YSM, & BMI	73, 74	100, 100	63.6, 61.3
16	Piedra et al^(^ ^36^ ^)^	2017	Prospective cohort	Spain	Healthy volunteer	261, 328	86, 80	61, 59
17	Beysel et al^(^ ^37^ ^)^	2019	Case control	Turkey	Age‐ & gender‐matched control	95, 60	80, 80	52.46, 52.5

Abbreviations: SI, Serial; PHPT, primary hyperparathyroidism; USA, United States of America; UK, United Kingdom; YSM, year since menopause.

^a^ Expressed as median.

### Primary outcome

**Fig 2:**
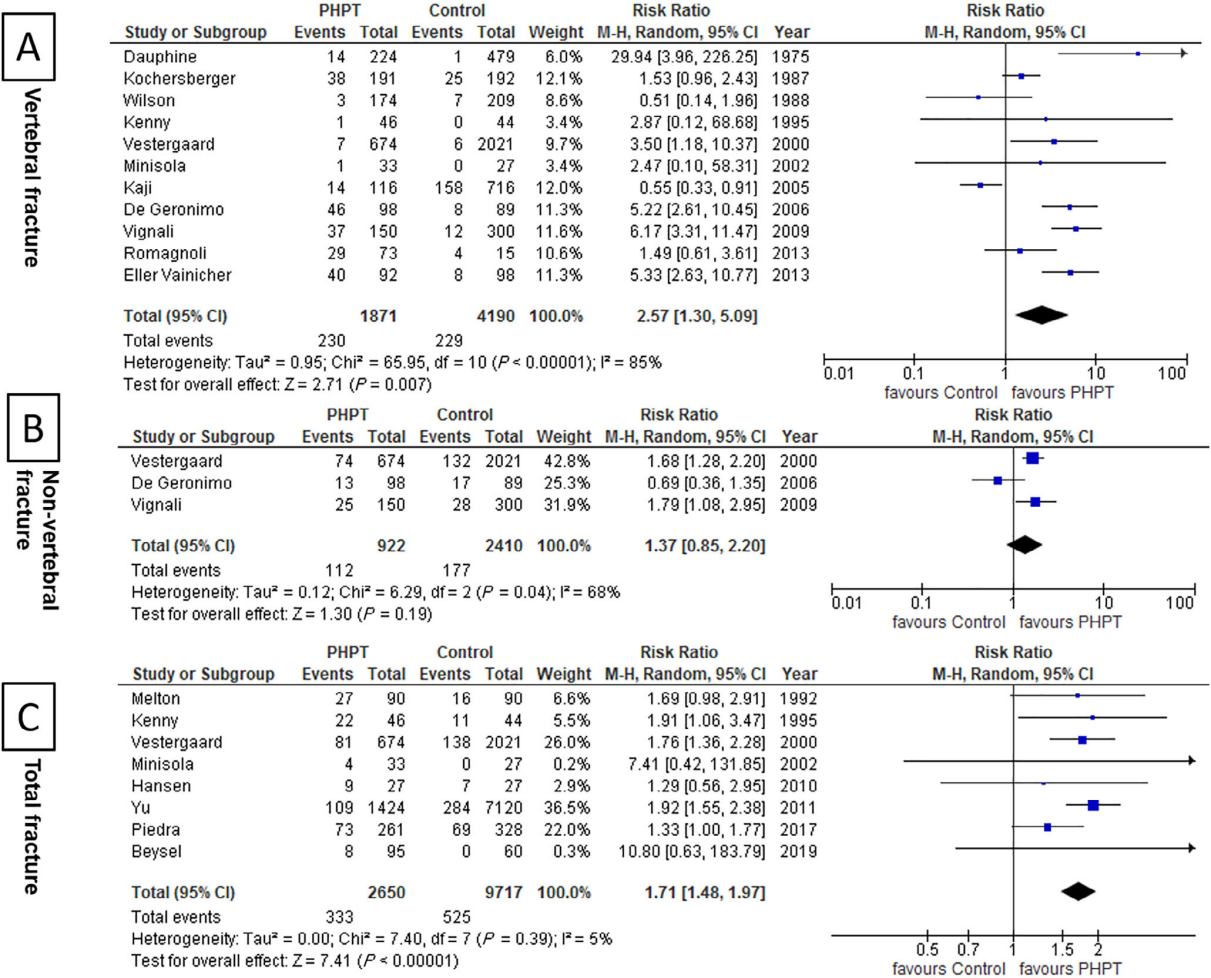
Forest plots showing primary outcomes: Comparison of fracture risk in patients with primary hyperparathyroidism (PHPT) versus controls: (*A*) vertebral fractures, (*B*) nonvertebral fractures, and (*C*) total fractures. M‐H, Mantel‐Haenszel.

Eleven studies^(^ ^21^, ^22^, ^23^, ^26^, ^27^, ^28^, ^29^, ^30^, ^31^, ^34^, ^35^ ^)^ had VF data for the PHPT population. The risk of VF was significantly higher among the patients with PHPT (RR, 2.57; 95% CI, 1.3–5.09; *p* = 0.007; Fig. 2*A*). Similarly, the risk of TF was higher (RR, 1.71; 95% CI, 1.48–1.97; *p* < 0.00001) among patients with PHPT compared with the control population (Fig. 2*C*). However, the risk of RF was not increased (RR, 1.94; 95% CI, 0.8–4.68; *p* = 0.14) among patients with PHPT (two studies).^(^ ^24^, ^27^ ^)^ Similarly, there was no difference in HF risk (RR, 1.45; 95% CI, 0.77–2.72; *p* = 0.25), but the data were available in only one study.^(^ ^27^ ^)^ Sensitivity analysis done for VF showed similar results after removing the studies by Wilson et al,^(23^ ^)^ which included only patients with mild PHPT, and by Romagnoli et al,^(^ ^35^ ^)^ which evaluated VF in a very small number of control patients. Similarly, the TF also did not change after removing the study by Yu et al, which included only patients with mild PHPT.^(^ ^33^ ^)^

Heterogeneity was nonsignificant for TF (*I* ^2^ 5%; *p* = 0.39), whereas it was high for VF (*I* ^2^ 85%; *p* < 0.00001) and NVF (*I* ^2^ 68%; *p* = 0.04). A funnel plot for publication bias did not show any visual asymmetry (Supplementary Information Fig. S1). Egger's and Begg's test also revealed no evidence of significant publication bias for VF (Egger's test, *p* = 0.44; Begg's test, *p* = 1.00), NVF (Egger's test, *p* = 0.5; Begg's test, *p* = 0.3), and TF (Egger's test, *p* = 0.45; Begg's test, *p* = 0.90). Publication bias could not be assessed for RF and HF because of a lesser number of studies.

### Secondary outcomes

**Fig 3:**
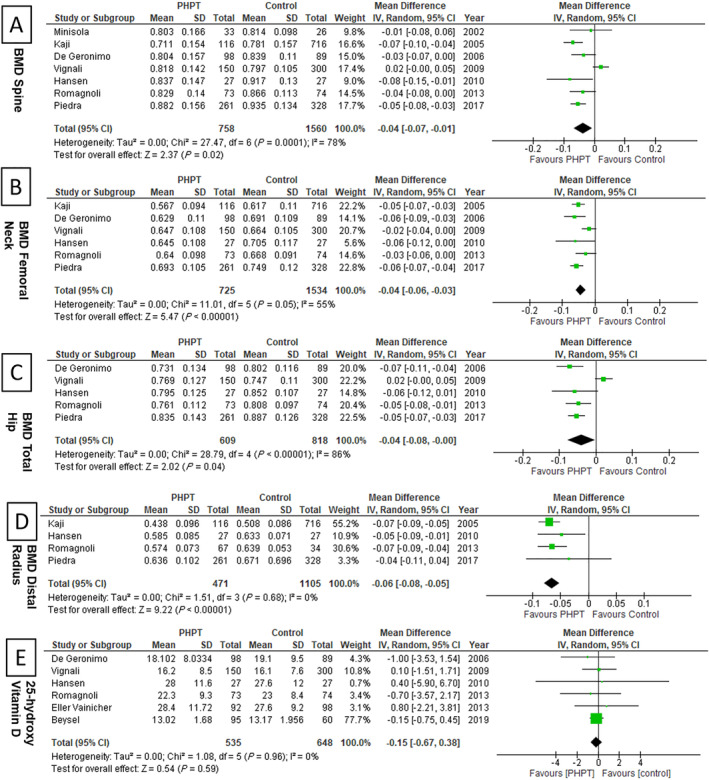
Forest plots showing secondary outcomes: Comparison of BMD and serum 25‐hydroxy vitamin D levels in patients with primary hyperparathyroidism versus controls: (*A*) spine, (*B*) femoral neck, (*C*) total hip, (*D*) distal radius, and (*E*) serum 25‐hydroxy vitamin D levels. IV, inverse variance.

In this meta‐analysis, seven studies^(^ ^26^, ^29^, ^30^, ^31^, ^32^, ^35^, ^36^ ^)^ compared BMD in patients with PHPT with the control population. All included patients and controls underwent DXA with a Hologic device. The pooled analysis reveals a significant decrease in mean BMD for patients with PHPT with a MD of −0.04 g/cm^2^ at the lumbar spine, femoral neck, and total hip, but a MD of −0.06 g/cm^2^ at the distal radius (Fig. 3*A*–*D*).

In the subgroup analysis of VF, the risk of fracture is higher among symptomatic patients with PHPT (RR, 4.21; 95% CI, 1.57–11.79; *p* = 0.004) in comparison with the control population (Supplementary Information Fig. S2*D*). However, the risk of NVF is lower in mild patients with PHPT (RR, 0.07; 95% CI, 0.01–0.49; *p* = 0.008) with respect to controls (Supplementary Information Fig. S3*B*). However, the risk of TF is higher in women (RR, 1.74; 95% CI, 1.08–2.81; *p* = 0.02) and patients with mild PHPT (RR, 1.93; 95% CI, 1.56–2.39; *p* < 0.00001; Supplementary Information Fig. S4*A*,*B*). The risk of TF and NVF among the subgroup of male patients with PHPT could not be analyzed because of the unavailability of data.

Three of the included studies^(^ ^29^, ^31^, ^35^ ^)^ analyzed possible risk factors for VF among the patients with PHPT. Age (MD, 6.34; 95% CI, 2.05–10.62) and year since menopause (MD, 6.01; 95% CI, 3.27–8.76) were higher among patients with VF (Supplementary Information Fig. S5*A*,*B*). The BMD at the lumbar spine (MD, −0.08; 95% CI, −0.12 to −0.05) and distal radius (MD, −0.05; 95% CI, −0.07 to −0.03) were lesser in patients with VF (Supplementary Information Fig. S6*A*,*D*).

## Discussion

In this meta‐analysis, we found that the risks of both VF and TF are higher in patients with PHPT compared with the control population. PHPT is characterized by an increase in the bone remodeling rate, mostly through the upregulation in the expression of RANKL, a cytokine essential for osteoclast formation, survival, and activity.^(^ ^8^ ^)^ Concomitantly, there is a parallel decrease in osteoprotegerin expression and bone formation. Thus, there is a net bone loss caused by unbalanced focal bone remodeling favoring resorption over formation within the bone remodeling unit.^(8^ ^)^ Moreover, chronic pancreatitis, though less commonly associated with PHPT, can also lead to poor bone health caused by nutritional deficiency.^(^ ^38^, ^39^ ^)^ Similarly, in this meta‐analysis, we also found lower BMD at all four sites in patients with PHPT with a greater involvement of the distal radius. Densitometry and histomorphometric analysis have previously shown preferential involvement of cortical bone in PHPT, which are compatible with the known increased actions of PTH on cortical bone as compared with cancellous bone.^(^ ^40^ ^)^ Studies have shown that weight‐bearing can mitigate the effect of PTH excess in the PHPT population.^(^ ^41^ ^)^ This may explain the relative preservation of hip BMD, a weight‐bearing site over radius BMD, a nonweight‐bearing site in our study, though both are cortical bone predominant sites.

As PHPT subjects have thinning of cortical bone with relative preservation of trabecular bone, an increased incidence of fractures at cortical but not trabecular sites would be anticipated.^(^ ^42^ ^)^ On the contrary, we found an increased risk of VF despite vertebrae having predominantly trabecular bone architecture. However, recent studies have challenged this notion and showed involvement of cancellous bone along with cortical bone in PHPT. In the study by Stein et al,^(^ ^43^ ^)^ HRpQCT showed the reduced volumetric densities at both cortical and trabecular compartments with thinner cortices and more widely spaced trabeculae. Trabecular bones have relatively fewer plate‐like trabeculae, reduced connectivity, and a less‐aligned trabecular network as determined by individual trabecular segmentation analysis of the HRpQCT images. These factors lead to the decrease in trabecular bone strength along with cortical bone in PHPT.^(^ ^43^ ^)^ Finite element analysis (FEA) of the HRpQCT images found lower whole‐bone and trabecular stiffness in PHPT than in controls.^(^ ^44^ ^)^ Similarly, trabecular bone scores (TBSs) have shown partially degraded bone structure in patients with PHPT. Silva et al^(^ ^45^ ^)^ found that although over half of the subjects presented with normal lumbar spine *T* scores by DXA, only 27% of subjects had normal TBS values. The TBS has also been found to be associated with VF in subjects with PHPT.^(^ ^35^ ^)^ PHPT is associated with high bone remodeling, which causes deposition of younger and hence less‐mineralized tissue as shown by a quantitative backscattered electron imaging technique.^(^ ^44^ ^)^ This, along with the reduced collagen crosslink ratio (as measured by Fourier‐transform infrared imaging) affects the mechanical properties of the bone matrix, leading to reduced stiffness.^(^ ^44^ ^)^ Reduced mineralization density and reduced collagen maturity may contribute to an increased rate of fracture. All of these probable mechanisms can explain our finding of the increased risk of VF in patients with PHPT.

Symptomatic patients with PHPT are at higher risk of VF caused by severe disease as found in a subgroup analysis in our study. As most of the women were postmenopausal, additional estrogen deficiency can explain the increased risk of TF in them.^(^ ^8^ ^)^ However, patients with mild PHPT showed an increased risk of TF and decreased risk of NVF in this meta‐analysis. These findings are paradoxical and can be explained by factors like higher risk of fall in patients with PHPT, selection criteria of control subjects, and small number of studies included in the analysis.^(^ ^2^ ^)^ As the hip and distal radius are rich in cortical bone and BMD is lower at these sites in our meta‐analysis, an increased risk of NVF, HF, and RF would have been expected. Cortical thinning through PTH‐mediated endosteal resorption is compensated for by PTH‐mediated periosteal apposition, leading to bone with increased cross‐sectional diameter. This increase in bone size provides biomechanical protection for the skeleton and may also explain the absence of higher risk of NVF, including HF and RF, despite reduction in BMD in our meta‐analysis.^(^ ^46^, ^47^ ^)^

In the RCT by Lundstam et al,^(^ ^48^ ^)^ the risk of VF in patients with mild PHPT who underwent parathyroidectomy was not significantly different from those who were on an observation arm. A recent meta‐analysis by Singh et al^(^ ^49^ ^)^ also evaluated the effect of parathyroidectomy in patients with PHPT in comparison with active surveillance. Postparathyroidectomy patients with PHPT showed significant improvement in BMD at the lumbar spine and the femoral neck as opposed to those on active surveillance.^(^ ^47^ ^)^ However, the effect of parathyroidectomy on the fracture risk is not homogenous. Although cohort studies showed reduction in fracture risk, the RCT failed to show any improvement in fracture risk in the parathyroidectomy group when compared with those who were observed.^(^ ^20^, ^48^, ^50^, ^51^ ^)^ This difference could be attributable to the differences in follow‐up time, age of subjects, or selection bias present in patients selected for parathyroidectomy.^(^ ^10^, ^49^ ^)^

In the present study, we evaluated various risk factors between patients with and without VF in patients with PHPT. Major risk factors associated with increased risk of VF in patients with PHPT were advanced age, years since menopause, and low BMD at lumbar spine and distal radius. Age is an independent predictor of fracture risk, and the combination of age and low BMD is important in determining fracture risk in patients with PHPT.^(^ ^52^, ^53^ ^)^ Similarly, longer duration since menopause is more likely to affect bone health. Lower BMD of the corresponding fracture site (like BMD of spine for VF), is an established risk factor in the prediction of fracture risk. Though reduced BMD in the distal forearm is common in patients with PHPT, it is not conventionally considered to be a risk factor in the prediction of VF. In our study, we found low BMD at the distal radius is also an independent risk factor for VF. Similarly, Vilayphiou et al^(^ ^54^ ^)^ reported poor bone strength of distal radius as assessed by FEA was associated with an increased risk of VF in subjects with postmenopausal osteoporosis.

Our study is the first meta‐analysis that analyzes the fracture risk in patients with PHPT compared with apparently healthy controls. However, this study has a few limitations. First, there is a scarcity of studies determining RF, NVF, and HF in patients with PHPT. Only one of the included studies reported HF; thus, the risk of femur fracture could not be evaluated properly. Second, we did not perform a subgroup analysis depending on type of study design because of the limited number of studies with case control and cross‐sectional design. Third, the majority of included studies were of less than the ideal study quality. Fourth, studies for VF and NVF were significantly heterogenous with *I* ^2^ 85% and 68%, respectively. Several factors, such as the type of studies, ethnicities, the selection of the patients and controls, and the methods to define VF may account for the heterogeneity. Moreover, as the included studies were published over a span of five decades, the diagnostic criteria, assay methodology, and classification of patients with PHPT based on severity were heterogeneous.

To conclude, patients with PHPT are at significantly higher risk for both VF and TF associated with low BMD in comparison with controls. Advanced age, increased duration since menopause, and lower BMD at the spine and distal radius are risk factors for VF.

## Author Contributions

All authors contributed to the study conception and design. The data collection, analysis, and interpretation were performed by NN, CM, RP, and SSK. All authors revised it critically for important intellectual content and approved the final manuscript. JS takes responsibility for the integrity of the data analysis.

## Conflict of Interest

The authors declare that there is no conflict of interest that could be perceived as prejudicing the impartiality of the research reported.

### Peer Review

The peer review history for this article is available at https://publons.com/publon/10.1002/jbm4.10482.

## Supporting information


**Appendix S1**: Supplementary Information
