# Supplementary material for: The Risk of Fractures in Primary Hyperparathyroidism: A Meta‐Analysis
Source: JBMR Plus. 2021 Mar 16;5(4):e10482. doi: 10.1002/jbm4.10482 (PMC8046118; doi:10.1002/jbm4.10482)

**Supplemental Table 1:** Search strategy used for database

| **Search item** | |
| --- | --- |
| 1 | Primary hyperparathyroidism |
| 2 | PHPT |
| 3 | 1 OR 2 |
| 4 | Fracture |
| 5 | 3 AND 4 |
| **Search Strategy** | |
| "hyperparathyroidism, primary"[MeSH Terms] OR ("hyperparathyroidism"[All Fields] AND "primary"[All Fields]) OR "primary hyperparathyroidism"[All Fields] OR ("primary"[All Fields] AND "hyperparathyroidism"[All Fields]) OR "PHPT"[All Fields]) AND ("fractur"[All Fields] OR "fractural"[All Fields] OR "fracture s"[All Fields] OR "fractures, bone"[MeSH Terms] OR ("fractures"[All Fields] AND "bone"[All Fields]) OR "bone fractures"[All Fields] OR "fracture"[All Fields] OR "fractured"[All Fields] OR "fractures"[All Fields] OR "fracturing"[All Fields] | |

**Supplemental Table 2:** Assessment of study quality (MINORS scale)

| Questions | Dauphine | Kochersberger | Wilson | Larsson | Melton | Kenny | Vestergaard | Minisola | Hiroshi Kaji | Simona De Geronimo | Edda Vignali | Hansen | Ning Yu | Cristina Eller-Vainicher | Elisabetta Romagnoli | Piedra | Beysel |
| --- | --- | --- | --- | --- | --- | --- | --- | --- | --- | --- | --- | --- | --- | --- | --- | --- | --- |
| A clearly stated aim | 2 | 1 | 1 | 2 | 2 | 1 | 2 | 2 | 2 | 2 | 2 | 2 | 2 | 2 | 1 | 2 | 2 |
| Inclusion of consecutive patients | 2 | 0 | 1 | 0 | 2 | 0 | 0 | 2 | 1 | 2 | 2 | 0 | 0 | 2 | 2 | 0 | 0 |
| Prospective collection of data | 2 | 0 | 0 | 2 | 0 | 0 | 0 | 2 | 1 | 2 | 2 | 2 | 0 | 2 | 2 | 2 | 2 |
| Endpoints  appropriate to the aim of the study | 2 | 2 | 2 | 2 | 2 | 2 | 2 | 2 | 2 | 2 | 2 | 2 | 2 | 2 | 2 | 2 | 2 |
| Unbiased assessment of the study endpoint | 0 | 1 | 0 | 0 | 0 | 0 | 0 | 0 | 0 | 0 | 1 | 0 | 0 | 1 | 0 | 0 | 0 |
| Follow-up period appropriate to the  aim of the study | 0 | 0 | 0 | 0 | 0 | 0 | 0 | 0 | 0 | 0 | 0 | 0 | 0 | 1 | 0 | 0 | 0 |
| Loss to follow up less than 5% | 0 | 0 | 0 | 0 | 0 | 0 | 0 | 0 | 0 | 0 | 0 | 0 | 0 | 2 | 0 | 0 | 0 |
| Prospective  calculation of the study size | 0 | 0 | 0 | 0 | 0 | 0 | 0 | 0 | 0 | 0 | 0 | 0 | 0 | 0 | 0 | 0 | 0 |
| An adequate control group | 2 | 2 | 2 | 1 | 2 | 1 | 2 | 1 | 2 | 2 | 2 | 2 | 2 | 2 | 2 | 2 | 2 |
| Contemporary groups | 2 | 2 | 0 | 2 | 2 | 1 | 2 | 2 | 1 | 2 | 2 | 2 | 2 | 2 | 2 | 2 | 2 |
| Baseline equivalence of groups | 1 | 1 | 0 | 2 | 1 | 2 | 2 | 2 | 1 | 2 | 2 | 2 | 2 | 2 | 2 | 1 | 2 |
| Adequate statistical analyses | 2 | 2 | 1 | 2 | 2 | 2 | 2 | 2 | 2 | 2 | 2 | 2 | 2 | 2 | 2 | 2 | 2 |
| Total Score | 15 | 11 | 7 | 13 | 13 | 9 | 12 | 15 | 12 | 16 | 17 | 14 | 12 | 20 | 15 | 13 | 14 |

**Supplemental figure 1:** Funnel plot for publication bias for primary outcomes: (A) Vertebral

fracture, (B) Non-vertebral fracture, (C) Total fracture


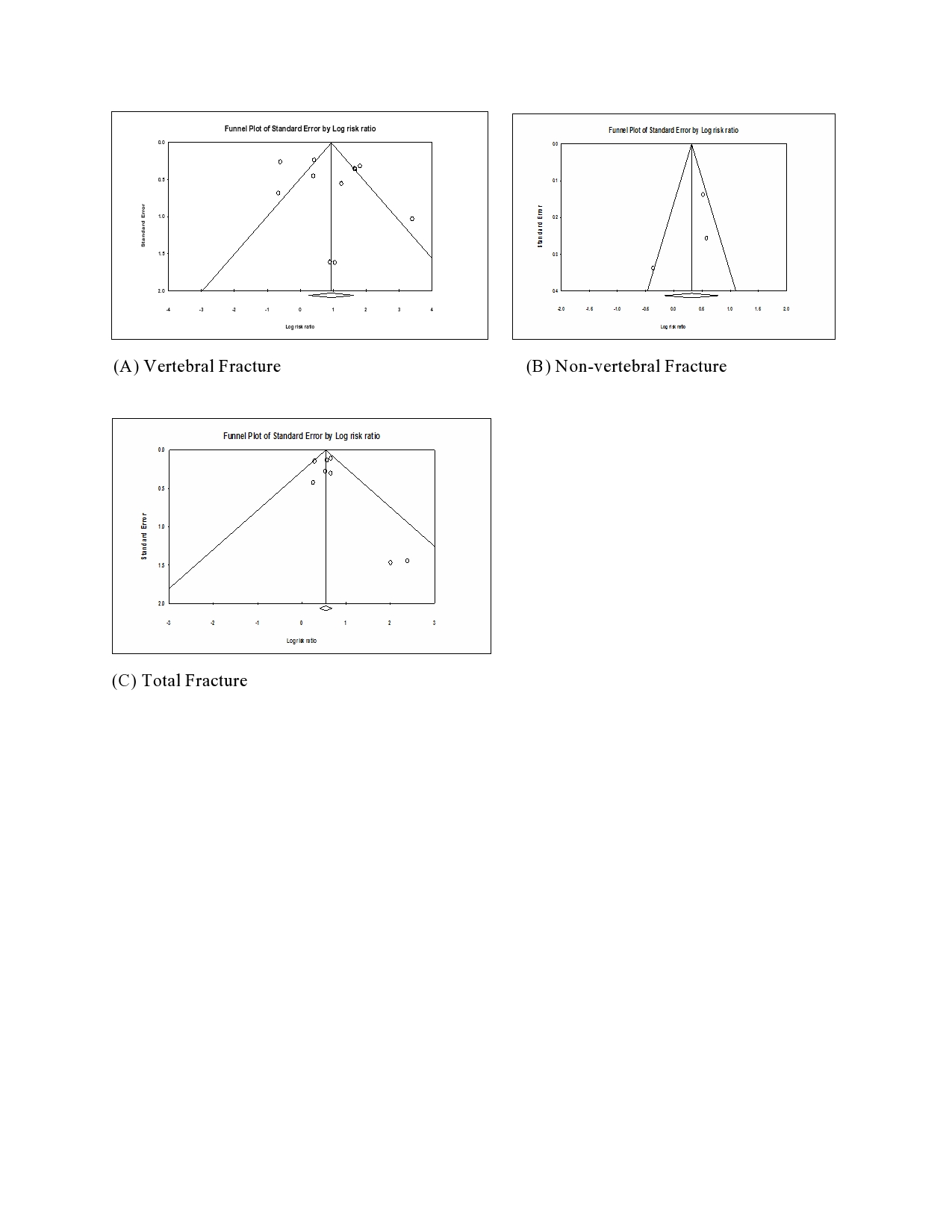


**Supplemental figure 2:** Subgroup analysis for risk of VF among the following groups of PHPT patients: (A) male, (B) Female, (C) Mild, (D) Symptomatic


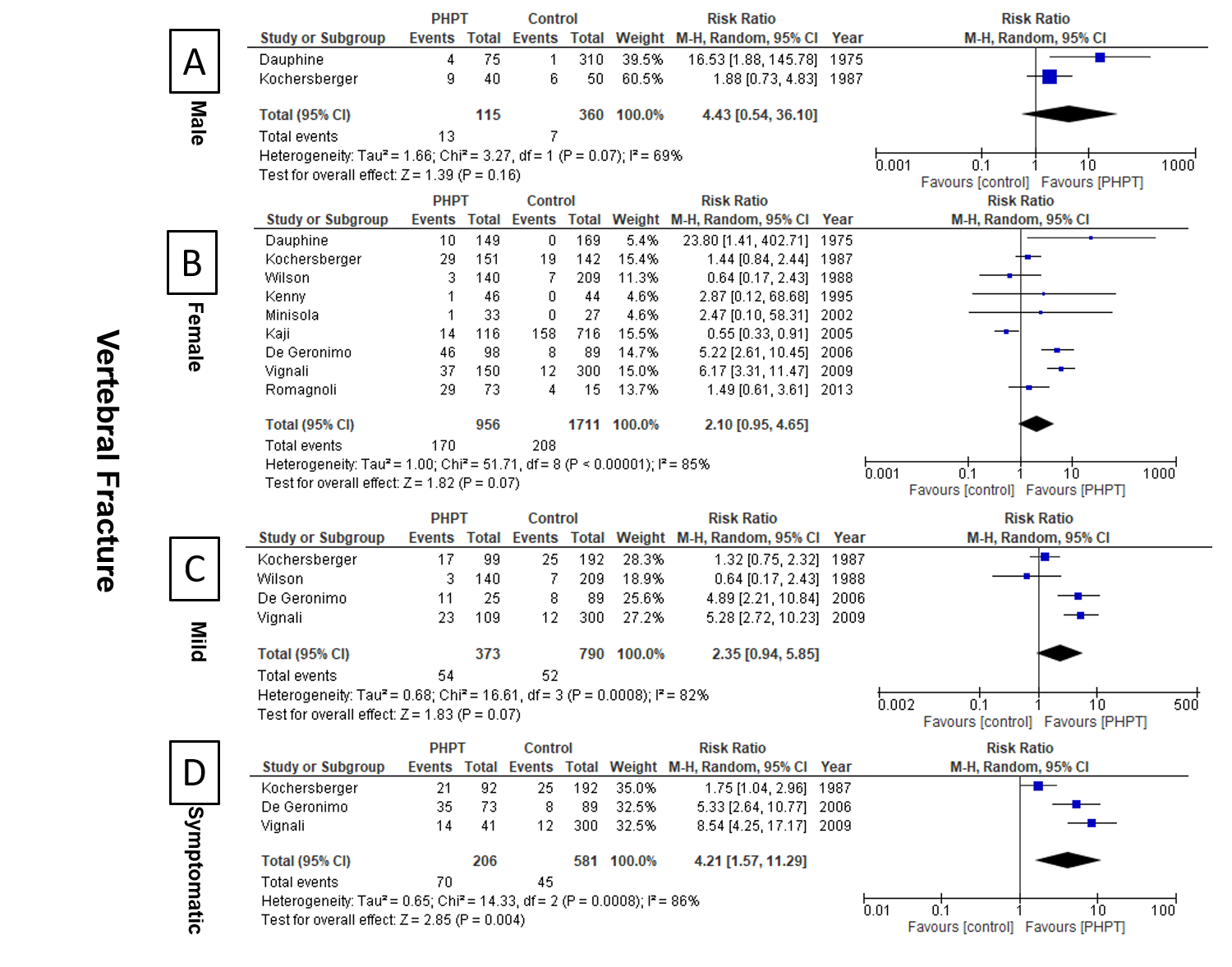


**Supplemental figure 3:** Subgroup analysis for risk of NVF among the following sub-groups of PHPT patients: (A) female, (B) Mild, (C) Symptomatic


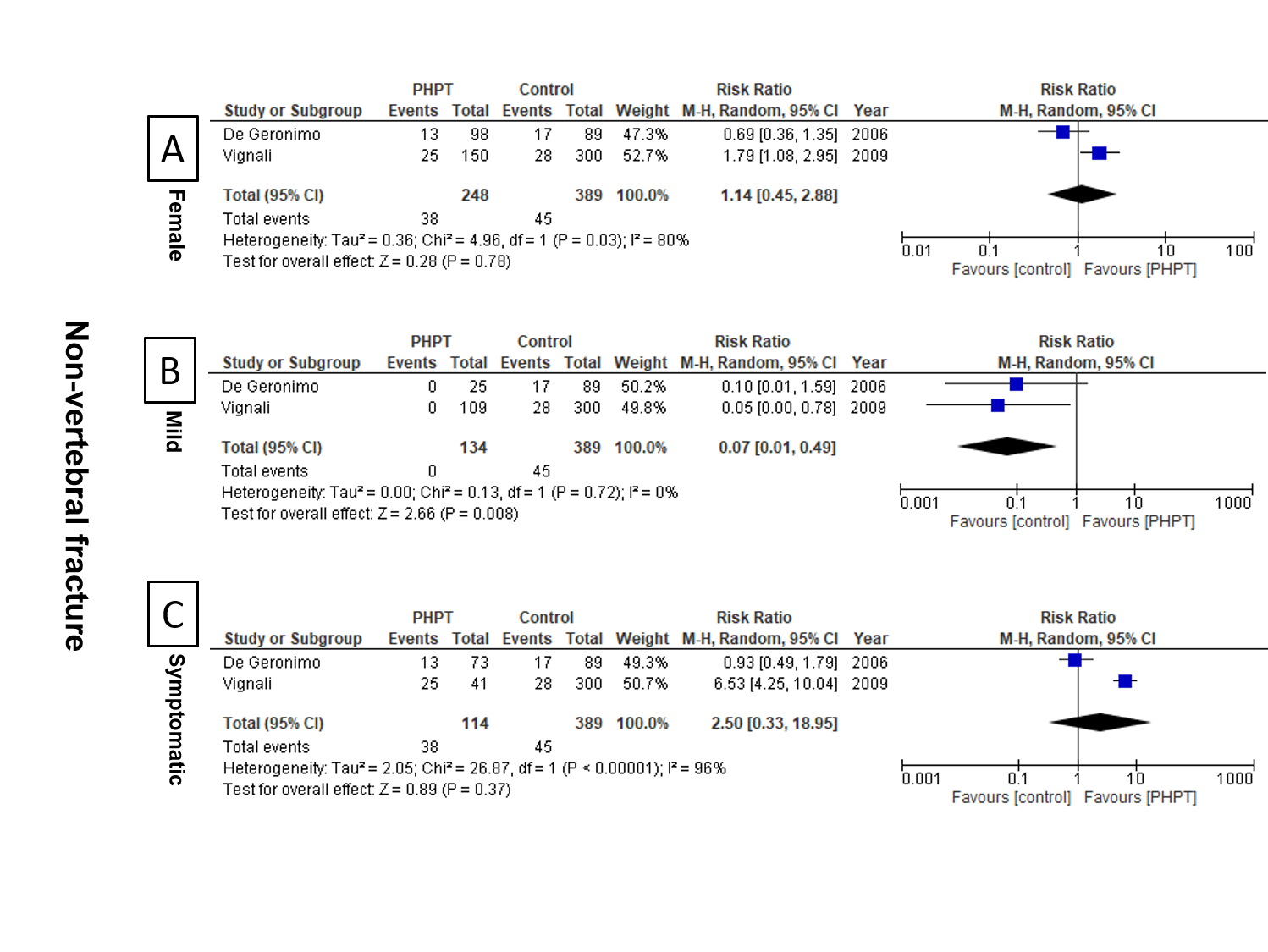


**Supplemental figure 4:** Subgroup analysis for risk of TF among the following sub-groups of PHPT patients: (A) female, (B) Mild


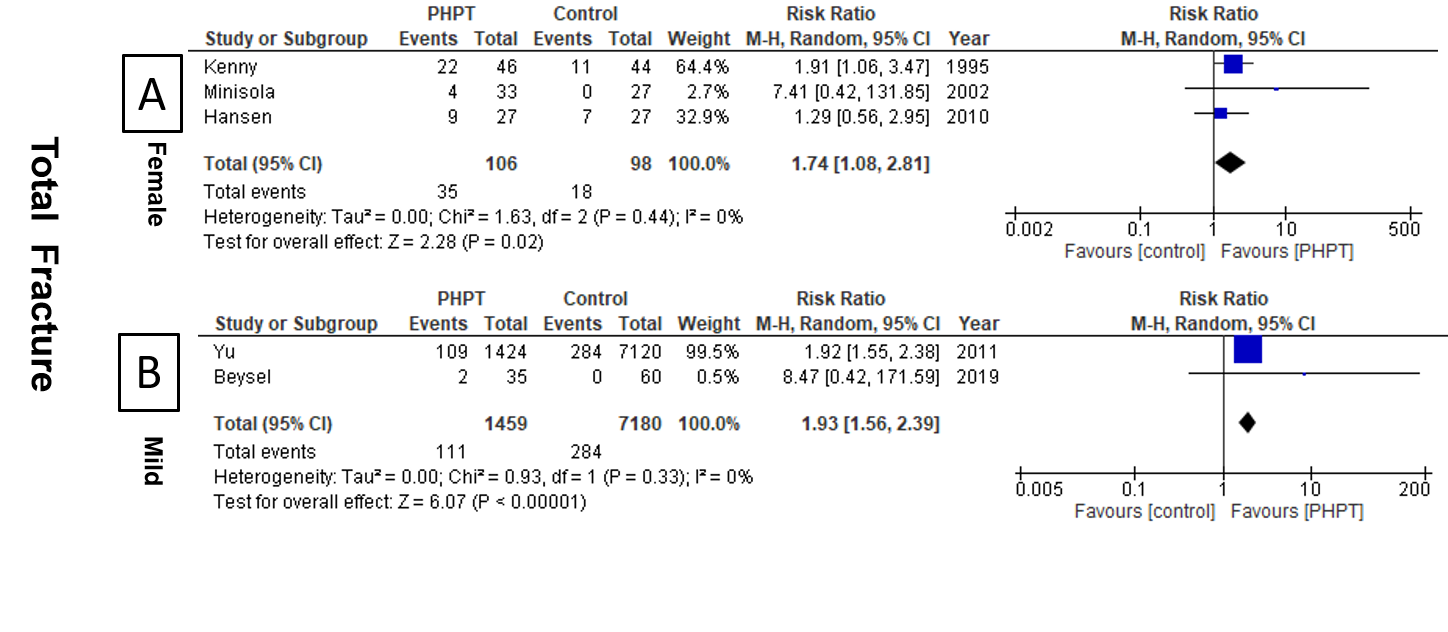


**Supplemental figure 5:** Forest plots comparing different factors among primary

hyperparathyroid patients with vertebral fracture (VF+) and without vertebral fracture (VF-):

(A) age, (B) year since menopause, (C) Body mass index (BMI), (D) PTH, (E) osteocalcin


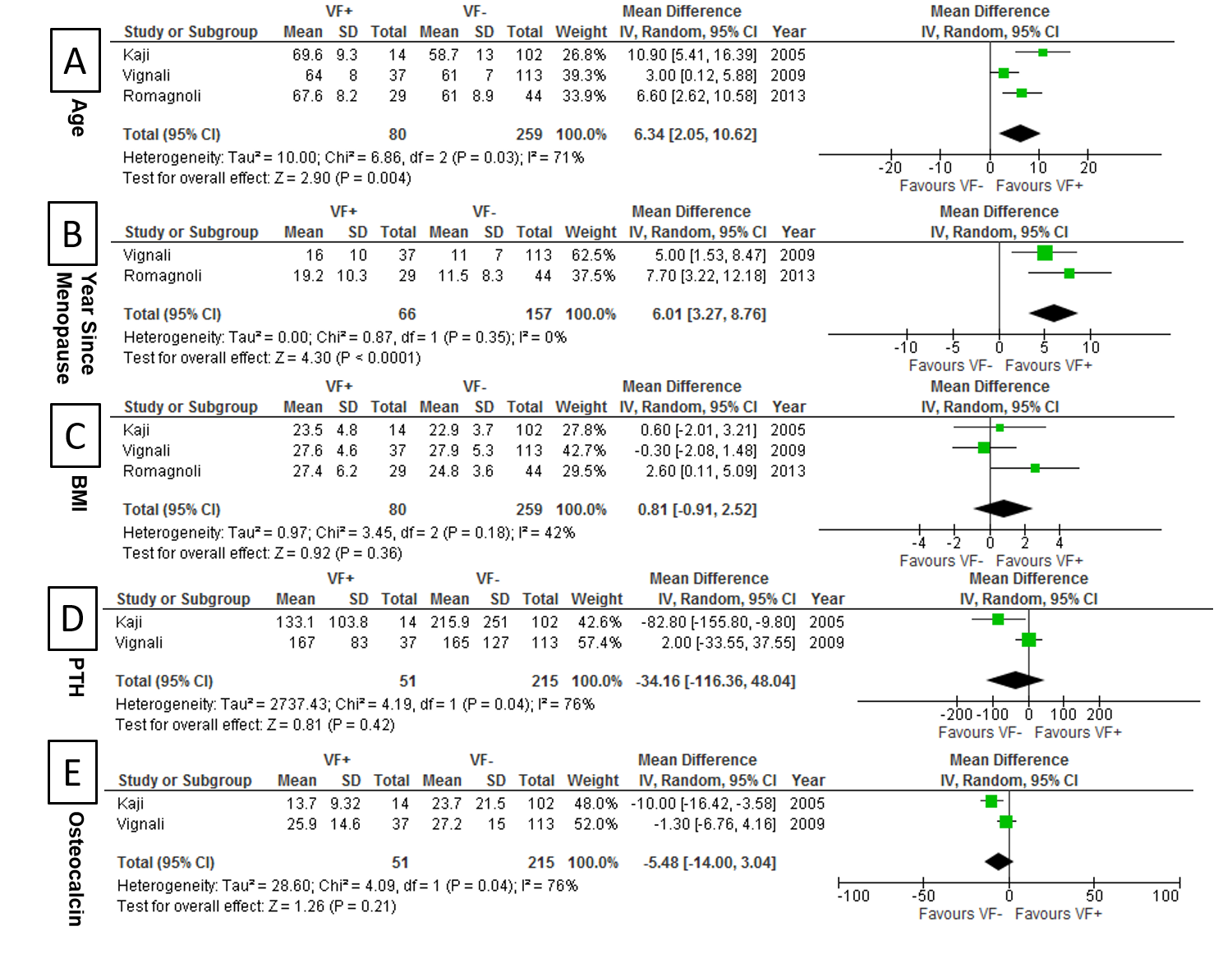


**Supplemental figure 6:** Forest plots comparing bone mineral density (BMD) among primary hyperparathyroid patients with vertebral fracture (VF+) and without vertebral fracture (VF-):

(A) Spine, (B) Femoral neck, (C) Total Hip, (D) Distal radius


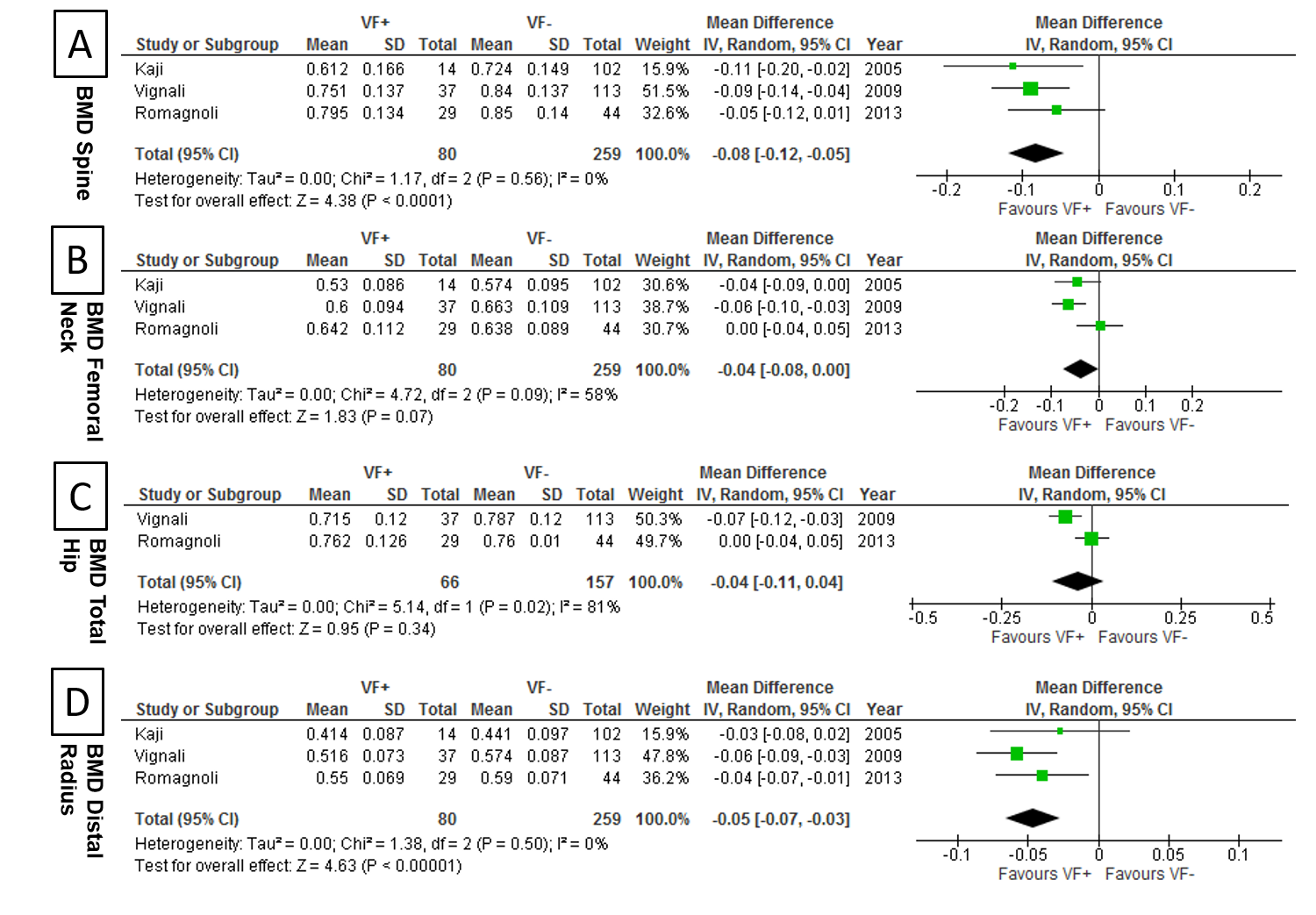

Supplement: Supplementary file 1 — Appendix S1: Supplementary Information [file JBM4-5-e10482-s001.docx]
